# Complete Genome Sequence of *Escherichia coli* Bacteriophage PGT2

**DOI:** 10.1128/genomeA.01370-17

**Published:** 2018-01-18

**Authors:** Alla K. Golomidova, Eugene E. Kulikov, Anna V. Kudryavtseva, Andrey V. Letarov

**Affiliations:** a Winogradsky Institute of Microbiology, RC Biotechnology RAS, Moscow, Russia; b Engelhardt Institute of Molecular Biology RAS, Moscow, Russia; c Department of Biology, Lomonosov Moscow State University, Moscow, Russia

## Abstract

Bacteriophage PGT2 was isolated from horse feces by using an uncharacterized *Escherichia coli* strain, 7s, isolated from the same sample as the host. Bacteriophage PGT2 and a related phage, phiKT, which was previously isolated from the same source, are likely to represent a new genus within the *Autographivirinae* subfamily of the *Podoviridae* family of viruses.

## GENOME ANNOUNCEMENT

Bacteriophages are ubiquitous components of all natural microbial systems (1), including the intestinal microbiomes of humans and animals. Microevolution is the way in which bacteriophages tend to adapt to their environment (2). *Escherichia coli* bacteriophages in the horse intestinal system are subjected to selective pressure driving the alterations and/or expansion of the phage host range in the course of short-term *in situ* evolution (3, 4). Therefore, comparative analysis of highly related and ecologically linked (e.g., obtained from the same animal or group of animals) phage isolates has become a valuable approach to deciphering the viral strategies of host cell recognition and infection.

Bacteriophage PGT2 was isolated from a sample of horse feces collected at an equestrian center at Neskuchny Garden, Moscow, Russia, in 2006. The isolate host was an uncharacterized environmental *E. coli* isolate, 7s, previously obtained from the same location (3). Transmission electron microscopy showed that PGT2 is a podovirus morphologically identical to phage phiKT, which was isolated from the *E. coli* 4s strain from horse feces collected at the same location. Phage phiKT uses the O22-type O antigen of its host (5) as its receptor molecule, but PGT2 did not grow on the 4s strain (5), while phiKT was not infective for the 7s *E. coli* isolate.

Bacteriophage PGT2 was grown and its DNA was extracted as described previously (3). The DNA was sequenced using a Roche 454 Junior sequencer, yielding 9,893 reads of an average length of 417 bp. Primary assembly was conducted with Newbler version 3.0, resulting in a single contig of 42,468 bp with a mean coverage of 96×. BlastN analysis with the NCBI online tool (https://www.ncbi.nlm.nih.gov) revealed that the nucleotide sequence of PGT2 was highly related to that of phiKT, with a total of 83% nucleotide identity, but most of the aligned regions show higher homology (92 to 97%). Both phages are very distantly related to coliphage T7. There are 9 major regions with low nucleotide homology between phiKT and PGT2, reflecting recent recombination events. Among them are the sequences encoding receptor recognition domains of the tail fibers. The receptor recognition domains of both phiKT and PGT2 are related to recently characterized domains of lateral tail fibers of T5-related phages that were also isolated from the same site (6), thus highlighting the importance of the characterization of ecologically linked phage isolates. Since the bacteriophages related to T7 have conserved sequences on the genome termini and possess 100- to 300-bp terminal repeats (TR), we set the ends of PGT2 at the same conserved sequences as those of phiKT. This yielded a TR length of 260 bp and total genome length of 42,686 bp. The genome end positioning is in agreement with that in our restriction fragment length polymorphism (RFLP) data. The genomic sequence of the PGT2 phage is considered a potential source of copyright-free protein sequences for biotechnological use.

Our analysis indicates that PGT2 should be classified as a new species within the *Autographivirinae* subfamily of *Podoviridae* according to the current International Committee on Taxonomy of Viruses (ICTV) classification (https://talk.ictvonline.org/taxonomy/). We also suggest the creation of a new genus, *phiKTvirus*, with the species phiKT and PGT2 within it.

### Accession number(s).

The draft genome sequence of bacteriophage PGT2 has been deposited in DDBJ/ENA/GenBank under the accession number MG201401.
